# (*E*)-*N*′-(5-Bromo-2-hy­droxy-3-meth­oxy­benzyl­idene)-2-hy­droxy­benzohydrazide monohydrate

**DOI:** 10.1107/S1600536812024816

**Published:** 2012-06-13

**Authors:** Shunsheng Zhao, Lanlan Li, Xiangrong Liu, Weixu Feng, Xingqiang Lü

**Affiliations:** a College of Chemistry and Chemical Engineering, Xi’an University of Science and Technology, Xi’an 710054, Shaanxi, People’s Republic of China; b College of Chemical Engineering, Northwest University, Xi’an 710069, Shaanxi, People’s Republic of China

## Abstract

The organic molecule of the title hydrate, C_15_H_13_BrN_2_O_4_·H_2_O, is roughly planar, with a mean deviation of 0.0939 (2) Å. The dihedral angle between the two aromatic rings is 8.2 (3)°. Intra­molecular O—H⋯N and O—H⋯O hydrogen bonds are observed. In the crystal, N—H⋯O(water) and O(water)—H⋯O hydrogen bonds lead to a three-dimensional network.

## Related literature

For related structures, see: Lu (2008 ▶); Nie (2008 ▶). For bond-length data, see: Allen *et al.* (1987 ▶). 
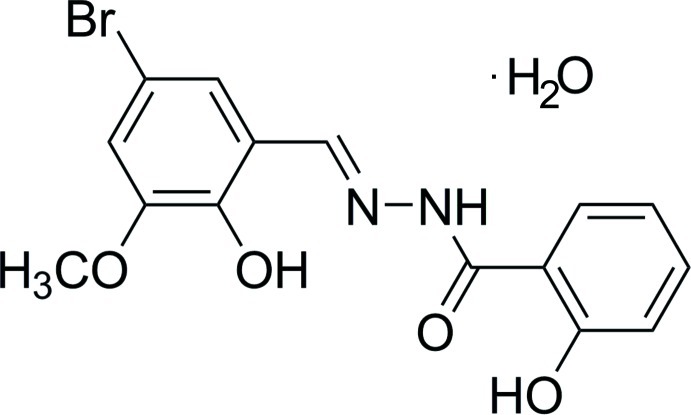


## Experimental

### 

#### Crystal data

C_15_H_13_BrN_2_O_4_·H_2_O
*M* *_r_* = 383.20
Orthorhombic, 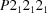

*a* = 6.3822 (13) Å
*b* = 14.142 (3) Å
*c* = 17.470 (4) Å
*V* = 1576.8 (6) Å^3^
*Z* = 4
Mo *K*α radiation
μ = 2.63 mm^−1^
*T* = 296 K
0.38 × 0.26 × 0.20 mm

#### Data collection

Bruker SMART 1K CCD area-detector diffractometer
Absorption correction: multi-scan (*SADABS*; Sheldrick, 2004 ▶) *T*_min_ = 0.444, *T*_max_ = 0.590
9336 measured reflections
3658 independent reflections
2060 reflections with *I* > 2σ(*I*)
*R*_int_ = 0.066

#### Refinement

*R*[*F* ^2^ > 2σ(*F* ^2^)] = 0.045
*wR*(*F* ^2^) = 0.131
*S* = 0.90
3658 reflections
217 parameters
3 restraints
H-atom parameters constrained
Δρ_max_ = 0.33 e Å^−3^
Δρ_min_ = −0.48 e Å^−3^
Absolute structure: Flack (1983 ▶), 1411 Friedel pairs
Flack parameter: 0.008 (15)

### 

Data collection: *SMART* (Bruker, 2001 ▶); cell refinement: *SAINT* (Bruker, 2001 ▶); data reduction: *SAINT*; program(s) used to solve structure: *SHELXS97* (Sheldrick, 2008 ▶); program(s) used to refine structure: *SHELXL97* (Sheldrick, 2008 ▶); molecular graphics: *SHELXTL* (Sheldrick, 2008 ▶); software used to prepare material for publication: *SHELXTL* and local programs.

## Supplementary Material

Crystal structure: contains datablock(s) I, global. DOI: 10.1107/S1600536812024816/fk2062sup1.cif

Structure factors: contains datablock(s) I. DOI: 10.1107/S1600536812024816/fk2062Isup2.hkl

Supplementary material file. DOI: 10.1107/S1600536812024816/fk2062Isup3.cml

Additional supplementary materials:  crystallographic information; 3D view; checkCIF report

## Figures and Tables

**Table 1:** Hydrogen-bond geometry (Å, °)

*D*—H⋯*A*	*D*—H	H⋯*A*	*D*⋯*A*	*D*—H⋯*A*
O2—H2*A*⋯N1	0.82	1.95	2.657 (5)	144
N2—H2*B*⋯O1*W* ^i^	0.86	2.09	2.921 (5)	164
O4—H4*A*⋯O3	0.82	1.80	2.527 (5)	147
O1*W*—H2*W*⋯O4^ii^	0.84	2.05	2.855 (6)	161
O1*W*—H1*W*⋯O3^iii^	0.85	2.06	2.823 (5)	148

Symmetry codes: (i) 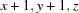; (ii) 
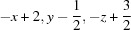
; (iii) 
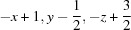
.

**Fig. 1.**
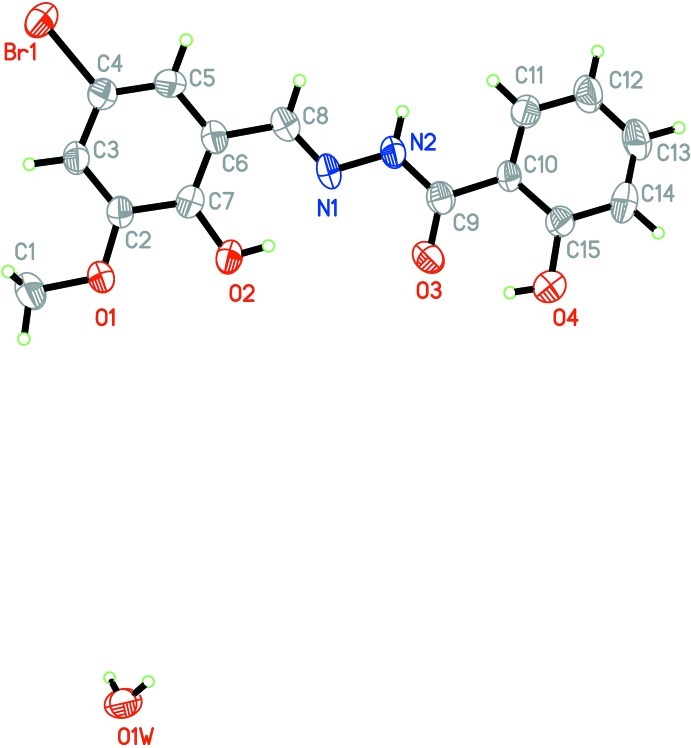
The molecular structure of the title compound with displacement ellipsoids drawn at the 50% probability level.

**Fig. 2.**
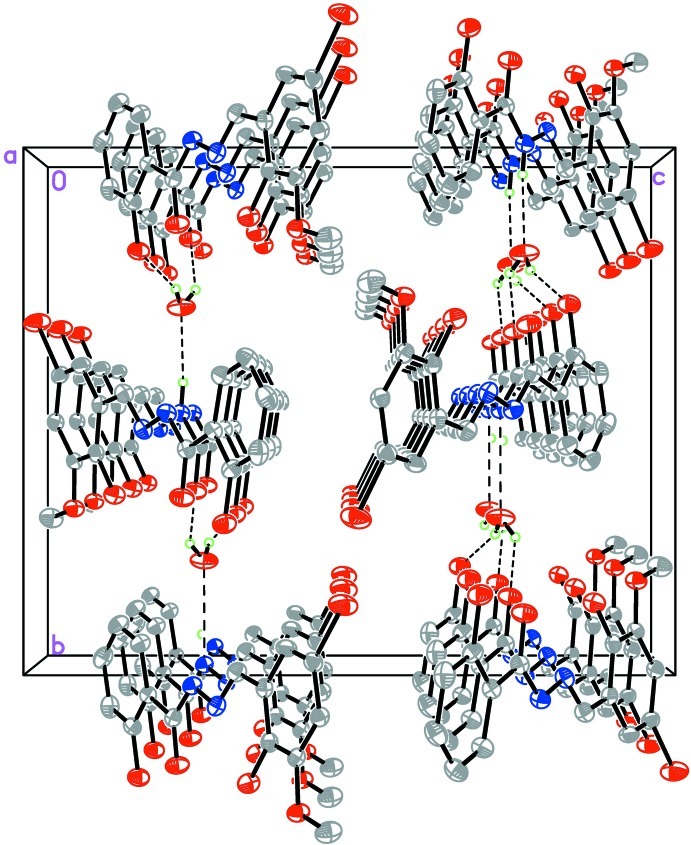
The crystal packing, viewed along the a axis, showing the hydrogen bonds as dashed lines.

## supplementary crystallographic information

### Comment

Hydrazones attract the interest of researchers due to their various biological activities *viz*. anticancer, anti-HIV, anthelmintic, antimycobacterial, anti-inflammatory, antidiabetic, antimicrobial, trypanocidal as well antimalarial activities. Here we report the crystal structure of the novel hydrazone title compound (Fig. 1). Bond lengths are in the range of expected values (Allen *et al.*, 1987) and are comparable to those observed from similar compounds (Lu, 2008; Nie, 2008). Intramolecular as well as intermolecular N—H···O and O—H···O hydrogen bonds are observed (Table 1), the intermolecular ones lead to a three-dimensional network of the hydrazone with water molecules (Fig. 2). The latter stems from non-dried acetonitrile.

### Experimental

2-Hydroxybenzohydrazide (152.2 mg, 1.0 mmol) was added to a solution of 5-bromo-2-hydroxy-3-methoxybenzaldehyde (231.5 mg, 1.0 mmol) in absolute ethanol (10 ml) and heated to reflux for 2 h. A pale yellow solid that precipitated from the reaction mixture was collected by filtration, dried on open air and then recrystallized from acetonitrile to give pale yellow crystals, yield 69.3%.

### Refinement

Hydroxy H atoms were located in a difference-Fourier synthesis and were refined as idealized rotating hydroxyl groups, with O—H = 0.82 Å and *U*_iso_(H) = 1.5 *U*_eq_(O). Water H atoms were located in a difference-Fourier synthesis and refined with constraint O—H = 0.82 Å, H···H distance 1.35 Å and *U*_iso_(H) = 1.5 *U*_eq_(O). Other H atoms were positioned geometrically and refined using a riding model with N—H = 0.86 Å, C—H = 0.93–0.96 Å and with *U*_iso_(H) = 1.2 (1.5 for methyl groups) times *U*_eq_(C/N).

### Crystal data

**Table:**

C_15_H_13_BrN_2_O_4_·H_2_O	*F*(000) = 776
*M**_r_* = 383.20	*D*_x_ = 1.614 Mg m^−^^3^
Orthorhombic, *P*2_1_2_1_2_1_	Mo *K*α radiation, λ = 0.71073 Å
Hall symbol: P 2ac 2ab	Cell parameters from 2451 reflections
*a* = 6.3822 (13) Å	θ = 1.9–26.6°
*b* = 14.142 (3) Å	µ = 2.63 mm^−^^1^
*c* = 17.470 (4) Å	*T* = 296 K
*V* = 1576.8 (6) Å^3^	Stick, colourless
*Z* = 4	0.38 × 0.26 × 0.20 mm

### Data collection

**Table:**

Bruker SMART 1K CCD area-detector diffractometer	3658 independent reflections
Radiation source: fine-focus sealed tube	2060 reflections with *I* > 2σ(*I*)
Graphite monochromator	*R*_int_ = 0.066
thin–slice ω scans	θ_max_ = 28.2°, θ_min_ = 1.9°
Absorption correction: multi-scan (*SADABS*; Sheldrick, 2004)	*h* = −8→5
*T*_min_ = 0.444, *T*_max_ = 0.590	*k* = −18→18
9336 measured reflections	*l* = −21→19

### Refinement

**Table:**

Refinement on *F*^2^	Secondary atom site location: difference Fourier map
Least-squares matrix: full	Hydrogen site location: difference Fourier map
*R*[*F*^2^ > 2σ(*F*^2^)] = 0.045	H-atom parameters constrained
*wR*(*F*^2^) = 0.131	*w* = 1/[σ^2^(*F*_o_^2^) + (0.0557*P*)^2^] where *P* = (*F*_o_^2^ + 2*F*_c_^2^)/3
*S* = 0.90	(Δ/σ)_max_ = 0.001
3658 reflections	Δρ_max_ = 0.33 e Å^−^^3^
217 parameters	Δρ_min_ = −0.48 e Å^−^^3^
3 restraints	Absolute structure: Flack (1983), 1411 Friedel pairs
Primary atom site location: structure-invariant direct methods	Flack parameter: 0.008 (15)

### Special details

**Table:**

Geometry. All e.s.d.'s (except the e.s.d. in the dihedral angle between two l.s. planes) are estimated using the full covariance matrix. The cell e.s.d.'s are taken into account individually in the estimation of e.s.d.'s in distances, angles and torsion angles; correlations between e.s.d.'s in cell parameters are only used when they are defined by crystal symmetry. An approximate (isotropic) treatment of cell e.s.d.'s is used for estimating e.s.d.'s involving l.s. planes.
Refinement. Refinement of *F*^2^ against ALL reflections. The weighted *R*-factor *wR* and goodness of fit *S* are based on *F*^2^, conventional *R*-factors *R* are based on *F*, with *F* set to zero for negative *F*^2^. The threshold expression of *F*^2^ > σ(*F*^2^) is used only for calculating *R*-factors(gt) *etc*. and is not relevant to the choice of reflections for refinement. *R*-factors based on *F*^2^ are statistically about twice as large as those based on *F*, and *R*- factors based on ALL data will be even larger.

### Fractional atomic coordinates and isotropic or equivalent isotropic displacement parameters (Å^2^)

**Table:**

	*x*	*y*	*z*	*U*_iso_*/*U*_eq_	
Br1	0.17855 (11)	1.17225 (4)	0.99142 (4)	0.0780 (3)	
O1	0.2998 (5)	0.8130 (2)	0.9293 (2)	0.0454 (9)	
O3	1.1945 (6)	0.8459 (2)	0.7425 (2)	0.0524 (10)	
O2	0.6469 (5)	0.8437 (2)	0.8561 (2)	0.0476 (9)	
H2A	0.7579	0.8596	0.8362	0.071*	
C6	0.6271 (8)	1.0123 (4)	0.8788 (3)	0.0370 (12)	
C10	1.4183 (7)	0.9660 (3)	0.6933 (3)	0.0343 (12)	
C3	0.2568 (8)	0.9790 (4)	0.9594 (3)	0.0392 (12)	
H3A	0.1357	0.9684	0.9878	0.047*	
N1	0.9280 (6)	0.9674 (3)	0.8055 (2)	0.0394 (11)	
C11	1.4638 (8)	1.0619 (4)	0.6803 (3)	0.0463 (14)	
H11A	1.3713	1.1075	0.6983	0.056*	
C8	0.8180 (8)	1.0329 (4)	0.8369 (3)	0.0431 (13)	
H8A	0.8621	1.0953	0.8327	0.052*	
C15	1.5614 (8)	0.9000 (4)	0.6654 (3)	0.0410 (13)	
C2	0.3633 (7)	0.9045 (4)	0.9263 (3)	0.0355 (11)	
C9	1.2306 (8)	0.9322 (4)	0.7355 (3)	0.0387 (12)	
N2	1.1036 (6)	0.9967 (3)	0.7668 (2)	0.0397 (11)	
H2B	1.1315	1.0560	0.7627	0.048*	
C14	1.7394 (8)	0.9297 (4)	0.6256 (3)	0.0521 (15)	
H14A	1.8342	0.8852	0.6071	0.063*	
O4	1.5336 (6)	0.8046 (2)	0.6757 (2)	0.0526 (10)	
H4A	1.4234	0.7953	0.6986	0.079*	
C4	0.3336 (9)	1.0699 (4)	0.9496 (3)	0.0431 (13)	
C7	0.5520 (8)	0.9207 (4)	0.8869 (3)	0.0377 (12)	
C13	1.7743 (9)	1.0233 (4)	0.6138 (3)	0.0546 (16)	
H13A	1.8916	1.0424	0.5863	0.066*	
C5	0.5154 (9)	1.0869 (4)	0.9111 (3)	0.0463 (14)	
H5A	0.5650	1.1484	0.9063	0.056*	
C1	0.1150 (8)	0.7915 (4)	0.9715 (4)	0.0597 (17)	
H1B	0.0880	0.7248	0.9688	0.090*	
H1C	−0.0015	0.8254	0.9501	0.090*	
H1D	0.1338	0.8098	1.0240	0.090*	
C12	1.6383 (9)	1.0900 (4)	0.6421 (3)	0.0520 (15)	
H12A	1.6656	1.1540	0.6351	0.062*	
O1W	0.1142 (6)	0.2032 (3)	0.7660 (3)	0.0683 (13)	
H2W	0.2215	0.2368	0.7723	0.102*	
H1W	0.0225	0.2390	0.7451	0.102*	

### Atomic displacement parameters (Å^2^)

**Table:**

	*U*^11^	*U*^22^	*U*^33^	*U*^12^	*U*^13^	*U*^23^
Br1	0.0908 (5)	0.0470 (3)	0.0962 (6)	0.0036 (3)	0.0521 (4)	−0.0121 (4)
O1	0.040 (2)	0.039 (2)	0.057 (2)	−0.0034 (18)	0.0170 (18)	0.0032 (17)
O3	0.041 (2)	0.041 (2)	0.075 (3)	−0.0072 (18)	0.0064 (19)	0.0060 (18)
O2	0.038 (2)	0.046 (2)	0.059 (2)	0.0063 (18)	0.0169 (18)	0.0016 (18)
C6	0.030 (3)	0.047 (3)	0.034 (3)	−0.004 (2)	0.002 (2)	0.000 (2)
C10	0.023 (3)	0.041 (3)	0.039 (3)	0.000 (2)	0.001 (2)	0.002 (2)
C3	0.035 (3)	0.044 (3)	0.039 (3)	−0.001 (2)	0.009 (2)	0.002 (2)
N1	0.026 (2)	0.050 (3)	0.042 (3)	−0.005 (2)	0.0054 (19)	0.007 (2)
C11	0.041 (3)	0.045 (3)	0.053 (4)	0.004 (3)	0.008 (3)	0.004 (3)
C8	0.035 (3)	0.047 (3)	0.047 (3)	−0.007 (3)	0.003 (3)	0.005 (2)
C15	0.034 (3)	0.043 (3)	0.046 (3)	−0.001 (3)	−0.001 (2)	−0.006 (3)
C2	0.031 (3)	0.040 (3)	0.036 (3)	0.001 (2)	0.003 (2)	0.003 (2)
C9	0.032 (3)	0.045 (3)	0.038 (3)	−0.001 (2)	−0.002 (2)	0.003 (2)
N2	0.031 (2)	0.042 (3)	0.046 (3)	−0.003 (2)	0.010 (2)	0.004 (2)
C14	0.036 (3)	0.074 (5)	0.046 (3)	0.008 (3)	0.009 (3)	−0.003 (3)
O4	0.049 (2)	0.043 (2)	0.065 (3)	0.0035 (18)	0.0088 (19)	−0.0034 (19)
C4	0.045 (3)	0.043 (3)	0.041 (3)	−0.005 (3)	0.008 (3)	−0.006 (2)
C7	0.035 (3)	0.044 (3)	0.034 (3)	0.004 (2)	0.002 (2)	−0.001 (2)
C13	0.043 (4)	0.068 (4)	0.053 (4)	−0.007 (3)	0.013 (3)	0.016 (3)
C5	0.054 (4)	0.034 (3)	0.051 (4)	−0.006 (3)	0.012 (3)	−0.005 (3)
C1	0.044 (3)	0.055 (4)	0.080 (4)	−0.007 (3)	0.018 (3)	0.007 (3)
C12	0.042 (3)	0.057 (4)	0.057 (4)	−0.002 (3)	0.013 (3)	0.013 (3)
O1W	0.046 (2)	0.040 (2)	0.119 (4)	0.0026 (18)	0.003 (2)	−0.005 (2)

### Geometric parameters (Å, º)

**Table:**

Br1—C4	1.899 (5)	C8—H8A	0.9300
O1—C2	1.356 (6)	C15—O4	1.372 (6)
O1—C1	1.424 (6)	C15—C14	1.396 (7)
O3—C9	1.249 (6)	C2—C7	1.406 (7)
O2—C7	1.357 (6)	C9—N2	1.337 (6)
O2—H2A	0.8200	N2—H2B	0.8600
C6—C7	1.389 (7)	C14—C13	1.358 (8)
C6—C5	1.392 (7)	C14—H14A	0.9300
C6—C8	1.451 (7)	O4—H4A	0.8200
C10—C15	1.394 (7)	C4—C5	1.363 (7)
C10—C11	1.405 (7)	C13—C12	1.374 (8)
C10—C9	1.486 (7)	C13—H13A	0.9300
C3—C2	1.381 (7)	C5—H5A	0.9300
C3—C4	1.386 (7)	C1—H1B	0.9600
C3—H3A	0.9300	C1—H1C	0.9600
N1—C8	1.286 (6)	C1—H1D	0.9600
N1—N2	1.373 (5)	C12—H12A	0.9300
C11—C12	1.357 (7)	O1W—H2W	0.8409
C11—H11A	0.9300	O1W—H1W	0.8548
			
C2—O1—C1	118.1 (4)	C9—N2—H2B	120.3
C7—O2—H2A	109.5	N1—N2—H2B	120.3
C7—C6—C5	119.3 (5)	C13—C14—C15	120.1 (5)
C7—C6—C8	121.9 (5)	C13—C14—H14A	120.0
C5—C6—C8	118.8 (5)	C15—C14—H14A	120.0
C15—C10—C11	117.0 (5)	C15—O4—H4A	109.5
C15—C10—C9	119.1 (5)	C5—C4—C3	121.7 (5)
C11—C10—C9	123.9 (5)	C5—C4—Br1	119.9 (4)
C2—C3—C4	118.8 (5)	C3—C4—Br1	118.4 (4)
C2—C3—H3A	120.6	O2—C7—C6	123.6 (5)
C4—C3—H3A	120.6	O2—C7—C2	116.5 (5)
C8—N1—N2	116.0 (4)	C6—C7—C2	119.8 (5)
C12—C11—C10	122.1 (5)	C14—C13—C12	120.7 (5)
C12—C11—H11A	118.9	C14—C13—H13A	119.6
C10—C11—H11A	118.9	C12—C13—H13A	119.6
N1—C8—C6	122.0 (5)	C4—C5—C6	120.2 (5)
N1—C8—H8A	119.0	C4—C5—H5A	119.9
C6—C8—H8A	119.0	C6—C5—H5A	119.9
O4—C15—C10	121.8 (5)	O1—C1—H1B	109.5
O4—C15—C14	117.7 (5)	O1—C1—H1C	109.5
C10—C15—C14	120.4 (5)	H1B—C1—H1C	109.5
O1—C2—C3	124.4 (4)	O1—C1—H1D	109.5
O1—C2—C7	115.4 (4)	H1B—C1—H1D	109.5
C3—C2—C7	120.2 (5)	H1C—C1—H1D	109.5
O3—C9—N2	121.0 (5)	C11—C12—C13	119.6 (6)
O3—C9—C10	120.8 (5)	C11—C12—H12A	120.2
N2—C9—C10	118.2 (4)	C13—C12—H12A	120.2
C9—N2—N1	119.3 (4)	H2W—O1W—H1W	106.2

### Hydrogen-bond geometry (Å, º)

**Table:**

*D*—H···*A*	*D*—H	H···*A*	*D*···*A*	*D*—H···*A*
O2—H2*A*···N1	0.82	1.95	2.657 (5)	144
N2—H2*B*···O1*W*^i^	0.86	2.09	2.921 (5)	164
O4—H4*A*···O3	0.82	1.80	2.527 (5)	147
O1*W*—H2*W*···O4^ii^	0.84	2.05	2.855 (6)	161
O1*W*—H1*W*···O3^iii^	0.85	2.06	2.823 (5)	148

Symmetry codes: (i) *x*+1, *y*+1, *z*; (ii) −*x*+2, *y*−1/2, −*z*+3/2; (iii) −*x*+1, *y*−1/2, −*z*+3/2.
